# Nitrogen fixation by diverse diazotrophic communities can support population growth of arboreal ants

**DOI:** 10.1186/s12915-022-01289-0

**Published:** 2022-06-09

**Authors:** Maximilian Nepel, Josephine Pfeifer, Felix B. Oberhauser, Andreas Richter, Dagmar Woebken, Veronika E. Mayer

**Affiliations:** 1 Department of Botany and Biodiversity Research, University of Vienna, Vienna, Austria; 2 Department of Microbiology and Ecosystem Science, Centre for Microbiology and Environmental Systems Science, University of Vienna, Vienna, Austria; 3 Centre for the Advanced Study of Collective Behaviour, University of Konstanz, Konstanz, Germany

**Keywords:** Ant-plant interaction, *Azteca* ant, *Cecropia* plant, Microbial patch, Diazotrophy, ^15^N_2_ tracer assay, *nifH* gene sequencing

## Abstract

**Background:**

Symbiotic ant-plant associations, in which ants live on plants, feed on plant-provided food, and protect host trees against threats, are ubiquitous across the tropics, with the *Azteca*-*Cecropia* associations being amongst the most widespread interactions in the Neotropics. Upon colonization of *Cecropia*’s hollow internodes, *Azteca* queens form small patches with plant parenchyma, which are then used as waste piles when the colony grows. Patches—found in many ant-plant mutualisms—are present throughout the colony life cycle and may supplement larval food. Despite their initial nitrogen (N)-poor substrate, patches in *Cecropia* accommodate fungi, nematodes, and bacteria. In this study, we investigated the atmospheric N_2_ fixation as an N source in patches of early and established ant colonies.

**Results:**

Via ^15^N_2_ tracer assays, N_2_ fixation was frequently detected in all investigated patch types formed by three *Azteca* ant species. Quantified fixation rates were similar in early and established ant colonies and higher than in various tropical habitats. Based on amplicon sequencing, the identified microbial functional guild—the diazotrophs—harboring and transcribing the dinitrogenase reductase (*nifH*) gene was highly diverse and heterogeneous across *Azteca* colonies. The community composition differed between early and established ant colonies and partly between the ant species.

**Conclusions:**

Our data show that N_2_ fixation can result in reasonable amounts of N in ant colonies, which might not only enable bacterial, fungal, and nematode growth in the patch ecosystems but according to our calculations can even support the growth of ant populations. The diverse and heterogeneous diazotrophic community implies a functional redundancy, which could provide the ant-plant-patch system with a higher resilience towards changing environmental conditions. Hence, we propose that N_2_ fixation represents a previously unknown potential to overcome N limitations in arboreal ant colonies.

**Supplementary Information:**

The online version contains supplementary material available at 10.1186/s12915-022-01289-0.

## Background

The parallel rise and diversification of ants and plants have favored intimate ant-plant associations, many of which are mutually beneficial for both partners [1–3]. Loose associations between ants and plants, in which plants secrete small volumes of nectar from easily accessible organs outside the flowers (extrafloral nectaries), are widespread [4] and involve many different species. Specialized ant-plant interactions are characterized by modified hollow plant structures for shelter—so called domatia—observed in around 700 vascular plant species [5]. Additionally, the plant is providing food in the form of extrafloral nectar [6] and/or protein and carbohydrate-rich food bodies [7–12]. In return, ants protect their host plants against herbivores, pathogens, and encroaching vegetation [13, 14], and the ant’s wastes provide nutrients [15–19]. The extraordinary abundance of ants in the rainforest canopies and their N demand have been extensively discussed in the literature. Arboreal ants are able to adapt to resource imbalance due to N-poor food sources, e.g., through fueling foraging activities [20] or N-recycling and upgrading non-essential to essential amino acids via gut bacteria [21]. Additionally, biological N fixation (BNF), a process performed by certain bacteria and archaea (diazotrophs) that makes atmospheric N_2_ biologically available, has been hypothesized to supply ants with N and facilitate their high abundance in the rainforest canopies [22]. Although potential diazotrophs were found in the gut of arboreal ants [23–25] and *Pseudomonas* bacteria isolated from an ant-plant association were shown to grow on N-free media [26], BNF activity associated with arboreal ants could not be shown yet [27].

One of the most widespread ant-plant mutualisms in the Neotropics is the *Azteca*-*Cecropia* interaction. Arboreal *Azteca* species (Dolichoderinae) spend their entire life in and on *Cecropia* (Urticaceae) trees [28, 29] and nest in the internodes of the hollow *Cecropia* stem while defending their host plant effectively against intruders [30–32]. As the ants do not leave the tree, they are solely reliant on host plant-located food sources, such as plant-provided food bodies and hemipteran honeydew. Like in many other ant-plant interactions across the tropics, well-defined, dark-colored “patches” built by the ants can be found in inhabited domatia [33–36]. The first patch is initialized by the foundress queen right after colonization [37]. As the plant grows and the colony size increases, the worker ants enlarge the nesting space by colonizing additional internodes and form further patches throughout the whole nesting space of an established *Azteca* ant colony [38]. The patches contain numerous bacteria (pers. observation and Fukuda et al. [26]), nematodes [39, 40], and ascomycete fungi [37, 38], which are likely transmitted vertically from the mother colony [37] and might serve as food [35]. Several observations suggest that these patches are essential to ant colonies in the *Azteca*-*Cecropia* association: (i) the colony-founding (“foundress”) queen inoculates a small pile of scratched-off plant tissue (parenchyma) from the domatia walls with a cocktail of introduced patch organisms before laying eggs and (ii) the larval food in the early colony stage seems to be supplemented with patch material [37].

As the initial patch substrate is parenchyma, which is rich in carbon (C) but low in nitrogen (N), additional N might be needed to support the initial growth of the patch organisms. Upon entering, *Azteca* foundress queens seal the domatium and raise their first brood in isolation (claustral colony founding) by metabolizing their own internal nutritional reserves [41]. Besides defecation onto the patch by the queen, potential N sources are limited; yet, an alternative and potentially unlimited N source could be BNF.

The notable biomass of diverse patch organisms despite low N content of the initial parenchyma substrate leads to the hypothesis that (I) C-dominated patches of foundress queens are supplemented with N by BNF. Due to N-containing organic waste that *Azteca* worker ants frequently deposit (pers. observation), we hypothesize that (II) BNF is less prevalent in patches of established ant colonies than in more recently founded colonies. As these ant-maintained patches are supposedly essential for colony development and patch particles are transmitted vertically [37], we further hypothesize that (III) the diazotrophic community compositions in early and established ant colonies are tightly associated with the ant species and therefore vary little across colonies of the same ant species. Accordingly, we also expect that (IV) the *nifH*-transcribing community is dominated by a few specialized taxa.

Our study assesses the significance of BNF in this mutualistic ant-plant association by quantifying N_2_ fixation activity in patches of different *Azteca* species and colony ages. Additionally, we identify potential diazotrophs in this system by analyzing the marker gene and transcripts for N_2_ fixation, the dinitrogenase reductase (*nifH*) [42, 43].

## Results

### N_2_ fixation activity detected in all investigated patch types

**Fig. 1:**
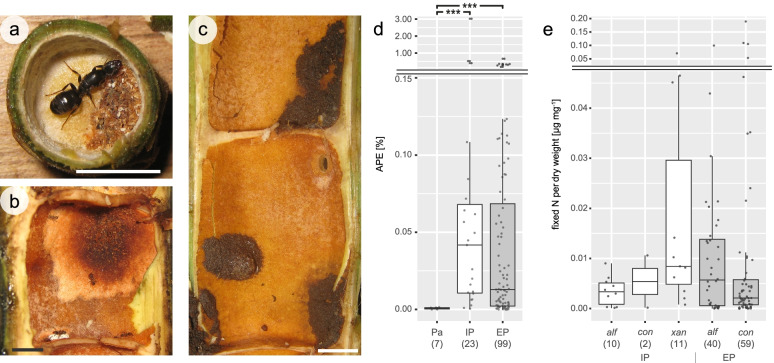
Visual appearance of patch types maintained by *Azteca* ants and quantified N_2_ fixation activity. **a** An initial patch (IP), here inside a cross-sectioned internode, is built up by a foundress queen scratching off parenchyma. Patch material of established **b** *A. alfari* and **c** *A. constructor* colonies (established patches (EP)) is consisting of plant material, dead nestmates, fungi, and nematodes. Patch samples and parenchyma from the inner walls of uninhabited plant internodes were incubated for 72 h in an artificial ^15^N_2_:O_2_ atmosphere. **d** The ^15^N atom percent excess (APE) [%] depicted on the *y*-axis shows significant N_2_ fixation in patches, but not in the plant parenchyma (Pa). **e** The amount of fixed N per mg sample (dry weight) depicted on the *y*-axis shows variations, but insignificant differences between *Azteca* species (*alf*, *A. alfari*; *con*, *A. constructor*; *xan*, *A. xanthochroa*) within patch types (IP, EP). Sample sizes are given in brackets, and significant differences are indicated by asterisks (***P < 0.001). Scale bars, 1 cm (**a**–**c**)

In order to evaluate the N_2_-fixing capability in the ant-plant *Cecropia*, we collected plant parenchyma, early patches built by foundress queens of the genus *Azteca* (“initial patch” (IP); Fig. 1a) and patch material from established *Azteca* colonies formed and maintained by worker ants (“established patch” (EP); Fig. 1b, c). Parenchyma and patches of three different *Azteca* species were incubated for 72 h in an artificial ^15^N_2_:O_2_ atmosphere, and subsequently, the ^15^N/^14^N isotopic ratios were determined. We found that ^15^N atom percent excess (APE) in patches was significantly higher than that in the parenchyma (Pa) from the walls of uncolonized plant internodes, in which ^15^N enrichment was not detectable (Pa vs. IP and Pa vs. EP: both *P* < 0.001; IP vs. EP: *P* = 0.118; Fig. 1d). In IPs, 0.011 μg N mg^−1^ dry weight (dw) was fixed on average, with a maximum of 0.068 μg N mg^−1^ dw. In EPs, 0.012 μg N mg^−1^ dw was fixed on average with a maximum of 0.189 μg N mg^−1^ dw. The quantity of fixed N did not vary significantly across ant species and patch types (between patch types: *P* = 0.28; between ant species within patch types: IP: *P* = 0.07; EP: *P* = 0.23; Fig. 1e). The overall mean BNF rate measured in *Azteca*-made patches was 3.9 μg N g^−1^ (dw) day^−1^.

### *Azteca* patches contain highly diverse diazotrophic communities

Aiming to assess the abundance of potential diazotrophs in patches of established colonies, we quantified the copy numbers of the functional marker gene for N_2_ fixation (*nifH*) and the 16S rRNA gene by quantitative PCR (qPCR) assays. The average proportion of diazotrophic to total microbial copy numbers per ng DNA of EPs was 3.2% and reached up to 14.7%. The ratios and abundance measures varied insignificantly between ant species (*nifH*: *P* = 0.786; 16S rRNA: *P* = 0.120; ratio: *P* = 0.349; Table 1).

**Table 1:** Quantitative abundances of microbial 16S rRNA and *nifH* gene copies in patches of established *Azteca alfari* and *A. constructor* colonies. Ratio depicts the proportions of diazotrophs to total microorganisms (*nifH* gene to 16S rRNA gene copy number). Values are displayed as the mean with standard error (SE)

Ant species	***nifH*** (copies ng^**−1**^ DNA)	SE	16S rRNA (copies ng^**−1**^ DNA)	SE	Ratio (%)	SE
*A. alfari*	3.40E+05	1.80E+05	2.50E+07	9.40E+06	2.8	1.38
*A. constructor*	1.30E+05	5.40E+04	5.00E+06	1.30E+06	3.6	0.95

**Fig. 2:**
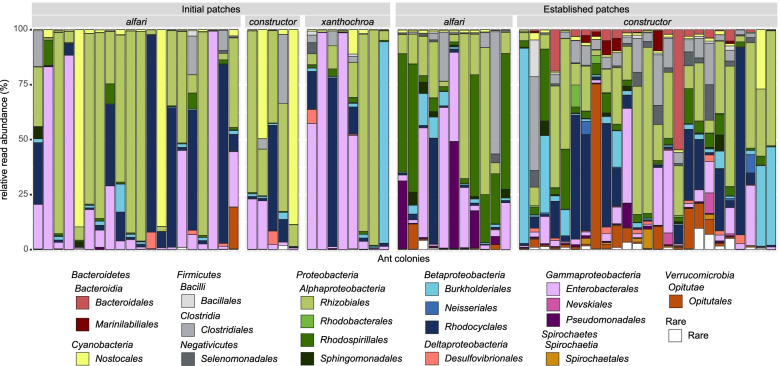
Diazotrophic community composition in *Azteca* ant-associated patch samples. Taxonomic assignments of OTUs are summarized on the order level based on BLASTP analysis. On the *x*-axis, every bar represents the diazotrophic community per ant colony, grouped by patch type (initial patch, established patch) and ant species (*Azteca alfari*, *A. constructor*, *A. xanthochroa*), whereas the *y*-axis shows the relative read abundance of taxonomic orders

To examine the diazotrophic communities in both ant-built patch types (IP and EP), we sequenced the *nifH* gene using Illumina MiSeq and processed the raw amplicon reads using the bioinformatic pipeline NifMAP [44]. Our data revealed a high diversity of diazotrophs, with a total of 1147 operational taxonomic units (OTUs) spanning eleven different phyla, based on the classification of *nifH* amino acid sequences. Across all patch samples, most reads were assigned to *Proteobacteria* (on average 81.3% of reads per sample), followed by *Firmicutes* (7.2%), *Cyanobacteria* (6.0%), *Verrucomicrobia* (3.0%), and *Bacteroidetes* (2.1%). The most abundant orders were *Rhizobiales* (on average 30.8% of reads per sample, *Alphaproteobacteria*), *Enterobacterales* (17.8%, *Gammaproteobacteria*), and *Rhodocyclales* (14.7%, *Betaproteobacteria*), though each ranged from 0% to more than 90% of relative read abundance amongst patches of individual ant colonies (Fig. 2). In accordance with this revealed heterogeneity in relative read abundances of bacterial orders, only 16 of 1147 *nifH* OTUs could be detected in 75% of the samples and only 6 of 74 genera were ubiquitous, namely *Bradyrhizobium*, *Azorhizobium* and *Rhodopseudomonas* (all *Rhizobiales*), *Azospirillum* (*Rhodospirillales*), *Paraburkholderia* (*Burkholderiales*), and *Klebsiella* (*Enterobacterales*). Neither, a single OTU nor genus, accounted constantly for more than 0.5% of reads per sample.

**Fig. 3:**
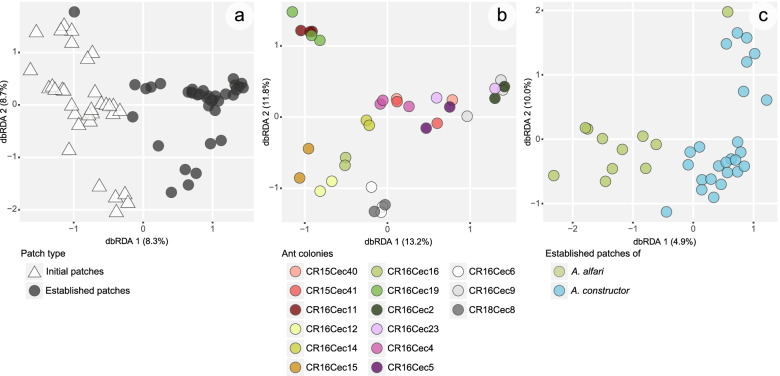
Distance-based redundancy analysis showing the variation of diazotrophic communities on OTU level **a** due to patch types, **b** within established patches due to individual ant colonies, and **c** within established patches due to ant species. Patch types are represented by shapes (IPs as triangles, EPs as circles). In **b**, multiple circles of the same color represent patch samples taken from different positions within the same plant

Despite the variations in relative read abundances of bacterial orders within IPs and EPs (Fig. 2, Additional file 1: Table S1), we observed distinct patterns in the diazotrophic community composition. IPs were mostly dominated by one of the abovementioned abundant orders, or by *Nostocales* (*Cyanobacteria*), while in EPs, other additional orders were highly abundant in single samples, e.g., *Rhodospirillales* (*Alphaproteobacteria*) and *Clostridiales* (*Firmicutes*) (Additional file 1: Table S1). IPs were overall significantly less diverse on the OTU level than EPs (Shannon index; on average: *A. alfari*, IP = 1.3, EP = 2.1, *P* = 0.021; *A. constr.*, IP = 1.9, EP = 2.9, *P* = 0.012; A. *xanthochroa*, IP = 1.2, Additional file 2: Fig. S1). The diazotrophic community composition of IPs and EPs displayed significant differences (*P* < 0.001, *R*^2^=0.083), visible in the constrained ordination (Fig. 3a). This was also reflected within ant species (IP vs. EP: *A. alfari*, *P* < 0.001, *R*^2^ = 0.102; *A. constr.*, *P* < 0.001, *R*^2^ = 0.079). Of several established ant colonies, patch material was sampled from up to three different locations throughout the nesting space representing different patch ages. Young patches were taken from recently colonized new domatia that develop apically during plant growth, while older patch material was sampled from lower parts of the ant’s nesting space. The position of the collected EPs within the plant, representing different patch age, did not affect the diazotrophic community composition (*P* = 0.994). Consistently, the EPs of the same ant colony harbored similar diazotrophic communities; thus, the most significant predictor for diazotroph community composition was the individual ant colony that inhabited one *Cecropia* tree (*P* < 0.001, *R*^2^ = 0.749, Fig. 3b). The individual *Cecropia* tree played an insignificant role, as in *Cecropia* saplings with multiple independent colonization events, the IP community compositions did not correlate with the plant ID (*P* = 0.084). Within patch types, the ant species significantly correlated with the community composition of EPs (*P* = 0.0029; *R*^2^ = 0.049; Fig. 3c), but not of IPs (*P* = 0.316).

**Fig. 4:**
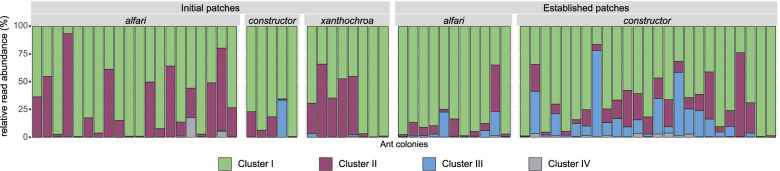
Relative abundance of phylogenetic *nifH* clusters based on classification and regression trees (CART) analysis. On the *x*-axis, every bar represents the diazotrophic community of an individual ant colony, grouped by patch type (initial patch, established patch) and ant species (*Azteca alfari*, *A. constructor*, *A. xanthochroa*), whereas the *y*-axis shows the relative read abundance

Ecophysiological traits of diazotrophs were inferred from *nifH* amino acid sequences, by assigning OTUs to one of four phylogenetic clusters based on regression trees (Fig. 4). In both patch types, most reads belonged to the phylogenetic cluster I (IP: on average 71% of reads per sample, EP: on average 72%), harboring mainly the canonical Mo-Fe nitrogenase of aerobic *Proteobacteria* and *Cyanobacteria*. A considerable percentage of reads was assigned to cluster II, which represents the alternative nitrogenase *anfH* using only Fe as a cofactor (IP 26%, EP 14%). Reads of cluster III consisting mainly of obligate anaerobic diazotrophs harboring the canonical Mo-Fe nitrogenase were almost exclusively present in patches of established ant colonies and more abundant in EPs of *A. constructor* than *A. alfari* (IP 1%, EP 12%; *alf* 5%, *con* 15%). The potentially inactive nitrogenases of cluster IV were hardly present in both sample types (IP 0.7%, EP 0.7%).

### High diversity of diazotrophs actively transcribing *nifH* genes

**Fig. 5:**
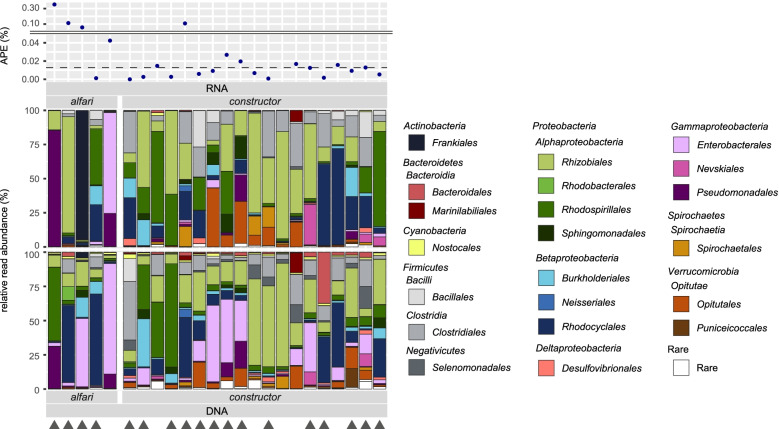
High heterogeneity of the *nifH*-transcribing community in patches of established *Azteca alfari* and *A. constructor* colonies at the time of sampling. Taxonomic assignments of OTUs are summarized on the order level based on BLASTP analysis. On the *x*-axis, every bar represents the diazotrophic community (DNA, bottom) and the actively transcribing community (RNA, middle) of one EP sample, grouped by ant species. The *y*-axis shows the relative read abundance of taxonomic orders. Triangles denote samples in which the most abundant transcribing order is not the most abundant one on the diazotrophic community level. The top graph shows the corresponding ^15^N atom percent excess (APE) after samples were incubated with ^15^N_2_. The dashed line denotes the median APE of all measured EP samples

To identify potential key diazotrophs that were actively expressing the *nifH* gene at the time of sampling, we sequenced reversely transcribed RNA (cDNA) of EPs. For both ant species, the actively *nifH*-transcribing communities were significantly less diverse than the *nifH* gene-harboring communities (Additional file 2: Fig. S2; mean Shannon indices; *A. alfari*: DNA 1.8, RNA 0.8, *P* = 0.036; *A. constr.*: DNA 2.9, RNA 2.1, *P* < 0.001). In Fig. 5, data of the *nifH* gene-harboring communities (based on DNA) are depicted together with the *nifH-*transcribing communities (based on RNA) derived from the same patch samples, along with their corresponding ^15^N APE after incubations. We recovered *nifH* transcripts from samples with varying N_2_ fixation activity, which was not associated with a particular taxonomic group. In general, reads assigned to *Alpha*-, *Beta*-, and *Gammaproteobacteria* were particularly abundant in actively *nifH*-transcribing communities of both ant species. However, no specific taxonomic group was ubiquitous and dominating these communities; instead, they were composed of diverse orders. Interestingly, in nearly all cases, the most abundant *nifH*-transcribing taxa at the time of sampling did not reflect the most abundant *nifH* gene-harboring taxa within the same samples (Fig. 5, denoted by triangles). This discrepancy was particularly apparent for *A. alfari*. In patches showing higher ^15^N enrichments than the median APE, reads assigned to the genera *Azospirillum* (*Rhodospirillales*), *Frankia* (*Frankiales*), *Kosakonia* (*Enterobacterales*), *Methyloferula* (*Rhizobiales*), and *Pseudomonas* (*Pseudomonadales*) were dominating the *nifH* transcripts of *A. alfari* EPs, whereas reads assigned to *Lachnoclostridium* (*Clostridiales*), *Rhodopseudomonas*, *Rhodomicrobium* (both *Rhizobiales*), *Magnetospirillum* (*Rhodospirillales*), *Sphingomonas* (*Sphingomonadales*), *Dactylopiibacterium* (*Rhodocyclales*), and an unclassified *Opitutaceae* (*Opitutales*) were most abundant amongst the *nifH* transcripts of *A. constructor* EPs (Additional file 2: Fig. S3).

## Discussion

### N_2_ fixation is a prevalent process in the *Azteca*-*Cecropia* symbiosis

In the domatia of their *Cecropia* host plant, *Azteca* ants create and maintain patches housing an intricate ecosystem, which might be crucial for colony survival. The observed abundance of patch organisms (fungi, nematodes, and bacteria) in C-dominated initial patches indicated the presence of additional N sources. Our study now provided the first evidence for atmospheric N_2_ fixation in ant-made patches, which can serve as an N source in arboreal *Azteca* ant colonies (Fig. 1). Via ^15^N_2_ tracer assays, we detected significant amounts of fixed N in initial patches (IPs) formed by foundress queens, thereby supporting our hypothesis that these patches obtain N via biological nitrogen fixation (BNF). This additional N presumably supports the growth of patch organisms in the C-dominated IP which, in turn, could help inhabiting ants raising their first larvae as their diet is supplemented with particles from IPs [37].

Contrary to our hypothesis, BNF was frequently detected and had similar rates in patches of established colonies (EPs) formed and maintained by worker ants as in foundress queen-initiated IPs. This is surprising, as these patches were frequently enriched by N-containing debris such as dead nestmates and occasionally food bodies (pers. observation), thus potentially decreasing the demand for BNF. In EPs of both ant species, the estimated proportion of diazotrophs to total bacteria was up to 14%, with an average of 3%, which is considerably higher than in various other terrestrial environments. For instance, in grassland or agricultural soil, as well as on plant surfaces and roots, diazotrophs have been estimated to account for less than 1% of the total microbial community [45–47]. This indicates selection in favor of BNF in patch ecosystems, potentially driven by the increasing demand for N due to increasing bacterial, fungal, and nematode biomass in later-stage patches.

### N_2_ fixation can support ant colony growth

The only previous evidence for ant-associated BNF activity was found in fungal gardens of ground-dwelling leaf cutter ants, which was estimated to account for around 50% of the garden’s N-demand based on natural isotope fractionation analysis [48]. While it is not possible to similarly model the N-input by BNF in *Azteca* colonies, BNF rates in fungal gardens of leaf cutter ants could be assessed based on measurements conducted by Pinto-Tomás and colleagues [48] (for detailed calculations, see Additional file 3: supplementary methods, Additional file 1: Table S2). The mean BNF rates we detected in the *Azteca* ant-built patches were around 200 times higher than those in the abovementioned fungal gardens, though the ^15^N_2_ incubation setup used for fungal garden pieces is believed to underestimate BNF activity (Pinto-Tomás, pers. communication). The mean BNF rate measured in *Azteca*-made patches (3.9 μg N g^−1^ (dw) day^−1^) also exceeded rates of other tropical habitats, such as canopy leaves, mosses, litter, and soil samples that range from appr. 0.001 to 2.8 μg N g^−1^ (dw) day^−1^ [49–52]. Only BNF rates of cyanolichens on volcanic soil in young forests and the highest detected rates in litter of a tropical lowland rainforest (up to 6 μg N g^−1^ (dw) day^−1^ [53, 54]) surpassed the mean BNF rate in ant-built patches but were still below the maximum rate (63.2 μg N g^−1^ (dw) day^−1^). Although the information on the life cycle and nutrition of these *Azteca* ant species is scarce, our approximation indicates that N provided by BNF can play a considerable role in ant population growth (for calculation details, see Additional file 3: supplementary methods). In established *A. alfari* and *A. constructor* colonies, up to 9.32 and 139.59 μg N, respectively, can be fixed daily, which correspond to approximately 66% and 900% of a worker ant’s N content. On average, it takes around 2 months from an egg to an adult ant [1, 55]. Under the assumption that all freshly fixed N ends up in *Azteca* larvae, the maximum N input extrapolated for a 2-month period corresponds to an “instant production” of up to 40 *A. alfari* and 598 *A. constructor* workers. Due to the small patch size of IPs, the amount of fixed N during the colony founding stage would be only sufficient for up to 0.5 workers, according to this calculation. Yet, its impact might be crucial for this most precarious colonization phase [56]. The first worker generation in claustral colony founding is typically miniature workers developed with the restricted nutrients provided solely by the queen [1]. Even putatively small amounts of additional N might lead to more workers or shorter development times and therefore could increase the chance of survival. In any case, BNF might enable the growth of patch organisms and thus facilitate the development of the diverse patch community including fungi, nematodes, and bacteria.

### Initial diazotrophic community is not shaped by the ant species

By scratching off plant parenchyma and forming the initial substrate for fungal cultivation [37], the queen is assumed to inoculate the C-dominated substrate with vertically transmitted bacteria from its mother colony, though very heterogeneous diazotrophic communities of various taxonomic orders dominated the *nifH* libraries of individual IP samples (Fig. 2). Contrary to our expectations, the diazotroph community composition in IPs was not shaped by the ant species. This does not confirm vertical transmission of diazotrophs from mother to daughter colonies; though, this also does not necessarily rule it out. As ascomycete fungi and nematodes were not only found in patches but also in the infrabuccal pockets of foundress queens when entering the plant internode [37], bacteria are likely transmitted vertically as well. The discovered diazotrophic heterogeneity within an ant species may be explained by two scenarios: the IP communities did not originate from the same mother colony, or varying environmental conditions and stochastic effects favor certain diazotrophs which further dominate patches in the early colony stage. Interestingly, despite detected taxa, e.g., *Rhizobiales* and *Enterobacterales* which are regularly reported as plant-associated diazotrophs, the host plants did not determine the diazotrophic community. The correlation between *Cecropia* saplings harboring multiple initial ant colonies and the diazotrophs was non-significant but had a low *p*-value. While this could indicate that some diazotrophs in patches are plant associated and were potentially introduced from the plant surface when the internode was entered, it might rather mirror that some initial ant colonies in one *Cecropia* plant originated from the same mother colony. Various environmental conditions can be expected to vary significantly between the plant surface and the patch ecosystem inside the ant nesting space (e.g., humidity, light intensity, temperature, nutrient availability, activity, and biomass of fungi, nematodes, and ants) implying a challenging environmental shift. Studying an increased number of *Cecropia* plants colonized by different *Azteca* species and investigating bacteria inside the infrabuccal pockets of foundress queens during their nuptial flight would be needed to finally clarify the role of the host plant and the mother colonies. *Rhizobiales*, *Enterobacterales*, and *Burkholderiales* have previously been hypothesized to be involved in recycling, upgrading, or fixing N_2_ in the gastrointestinal tract of arboreal ants [21, 23, 24, 57, 58], though most genera found in ant guts were not present in studied patches. Only *Klebsiella* and *Pantoea* (both *Enterobacterales*) were widely distributed and have been found in ant guts [23, 57], fungal gardens of leaf cutter ants [48], and now also in patches of *Azteca* colonies.

### Mature diazotrophic community differs from initial patches and is associated with the ant species

Interestingly, the diazotrophic community composition changed during colony development and differed significantly between IPs and EPs (Fig. 3a), even though both patch types displayed similar BNF rates. The change in community composition when transitioning from IPs to EPs could be explained by multiple factors: First, by the colonization history, as it is common for *Azteca*-*Cecropia* and various other ant-plant associations [37, 59–62] that one plant houses several colony foundress queens in spatially separated domatia. As only one young ant colony takes over the *Cecropia* plant, several initial patch communities are finally fused and expected to increase microbial diversity. Second, new diazotrophs could be introduced from outside when ant workers in established *Azteca* colonies are patrolling on the surface of the *Cecropia* tree [63]. Third, the difference between diazotrophic communities in IPs and EPs could be due to the patch transformation during ant colony growth. The patch texture changes from brittle IPs (Fig. 1a) to extensive lawn-like EPs in *A. alfari* colonies (Fig. 1b) and dense voluminous EPs in *A. constructor* (Fig. 1c). Different textures will create variable environmental conditions, which could lead to changes in the diazotrophic community composition.

The diazotrophic community composition in EPs from different internodes of the same ant colony did not vary significantly. Our data imply that newly built EPs of subsequently colonized apical plant internodes are not randomly colonized by diazotrophs, but that worker ants must transfer EP material from existing patches as an inoculum onto new patches. The variation in diazotroph community composition correlated significantly with ant species, thereby supporting our hypothesis that the diazotrophs in established ant colonies are associated with the ant species (Fig. 3c). Differences in the community composition between EPs of both ant species could be caused by differing environmental conditions in these EPs. For instance, we identified a strong O_2_ gradient towards anoxia in EPs of *A. constructor*, whereas in *A. alfari* EPs, only atmospheric O_2_ concentrations were measured (Additional file 2: Fig. S4, Additional file 3: supplementary methods). Reduced O_2_ concentrations could offer niches for potentially anaerobic diazotrophs (e.g., cluster III, Fig. 4) and are reflected in the higher relative abundance of mainly anaerobic bacterial groups such as *Bacteroidales*, *Clostridiales*, and *Opitutales* in *A. constructor* compared to *A. alfari* EPs (Fig. 2, established patches).

### Heterotrophic *nifH*-transcribing community implies resilience towards variable conditions

As BNF is a highly regulated process [64], sequencing of *nifH* genes merely identifies the community harboring these genes, while sequencing the *nifH*-transcripts reflects the diazotrophic community that actively expressed the genes and thus was likely involved in fixing atmospheric N_2_. Interestingly, in the majority of samples, the most abundant taxonomic order in DNA-based libraries did not coincide with the most abundant order in RNA-based libraries at the time of sampling (Fig. 5, denoted by triangles). This underlines the importance in investigating functional guilds beyond the DNA level, as actively transcribing community members cannot be inferred based only on the *nifH*-gene harboring community composition. We hypothesized that *nifH*-transcribing communities are dominated by few specialized taxa actively expressing the *nifH* gene, and indeed, the detected *nifH*-transcribing communities were less diverse than the *nifH* gene-harboring communities (Additional file 2: Fig. S2). However, there was no ubiquitous taxonomic group dominating all samples. Instead, in patches showing higher ^15^N enrichments than the median APE, we observed an unspecific active diazotrophic community, whose transcripts belonged mainly to twelve different taxonomic genera of nine orders (Additional file 2: Fig. S3). Amongst others, we found in situ *nifH* transcription of the genus *Pseudomonas* in investigated patches, thus supporting a recent study on the BNF capability of *Pseudomonas* isolates originating from an *Azteca*-*Cecropia* association in Brazil [26]. The genera *Klebsiella* and *Pantoea*, which are supposed to contribute to BNF in fungal gardens of leaf cutter ants [48] could be detected in DNA-based libraries, but not within the *nifH* transcripts.

## Conclusions

Our study provides the first evidence for atmospheric N_2_ fixation in ant-made patches located in the nesting space of the host trees—a so far overlooked potential N source for arboreal ants to overcome N limitations. Future research will show if this phenomenon is exclusive to the *Azteca-Cecropia* or can be found in other ant-plant associations. Although the sources of the diazotrophs serving as inoculum for freshly formed IPs need further investigation, the diverse and heterogeneous diazotrophic communities across ant colonies imply functional redundancy in ant-built patches, which could provide the ant-plant-patch system with a higher resilience towards changing environmental conditions like nutrient shortage, but also towards changes in substrate or O_2_ concentrations. Our data indicate that BNF can result in reasonable amounts of N especially in established ant colonies. This might not only enable bacterial, fungal, and nematode growth as well as community development in the patch ecosystems but can even support the growth of ant populations. Even though larval food seems to be supplemented with patch material during the early stage of a colony’s life cycle, more information on the nutrient fluxes inside the domatia and ants’ population dynamics are needed to confirm our indications that BNF is of nutritional relevance for *Azteca* population growth during early foundation and in established ant colonies.

## Methods

### Study site characteristics and sample collection

*Azteca*-colonized *Cecropia* trees were investigated between 2015 and 2018 in lowland areas of the Golfo Dulce region in SW Costa Rica close to the Tropical Research Facility La Gamba (www.lagamba.at; N08° 42′ 03″, W083° 12′ 06″, 70 m asl). Sampled *Cecropia* trees were collected from disturbed habitats at the margins of primary and secondary rain forests, alongside rivers (Rio Bonito, Rio Sardinal) and roads. *Azteca*-colonized *Cecropia* trees were cut in the field, wrapped in plastic bags, and transported to the research station. Colonies were categorized into two colonization stages, an *early founding stage* limited to one inhabited plant internode and an *established ant colony stage* with at least two internodes being inhabited. In the founding stage, solely the ant queen, possible brood, and an initial pile of scratched-off plant parenchyma (“initial patch” (IP)) were present. The entrance hole created by the founding queen to enter the hollow stem internode was always sealed with chewed plant tissue [37]. Established ant colonies, in contrast, reopened the entrance and connected additional internodes, in many of which they also formed clearly defined dark-colored patches (“established patches” (EPs)). After opening *Cecropia* stems, we collected patch material using sterile tweezers. Queens and worker ants (if present) were preserved in ethanol and classified using an *Azteca*-specific key [65]. We investigated IPs of *A. alfari* [Emery], *A. constructor* [Emery], and *A. xanthochroa* [Roger] and EPs of *A. alfari* and *A. constructor*, as no established *A. xanthochroa* ant colony inhabited one of our sampled *Cecropia* trees.

### Quantifying N_2_ fixation activity

For proof of N_2_ fixation activity in ant-built patches, a ^15^N_2_ tracer assay was used [66]. Fifty-six *Cecropia* plants were investigated, and in total, 122 samples were incubated in 2-mL glass vials containing an artificial ^15^N_2_:O_2_ (80:20) atmosphere. Early colony stage IPs (total *n* = 23; per species: *A. alfari* (10), *A. constructor* (2), *A. xanthochroa* (11)) were transferred directly from the internodes into the glass vial. EPs per established ant colonies (total *n* = 99; per species: *A. alfari* (36), *A. constructor* (59)) were homogenized first on a weighing paper before transferring parts into the glass vial for incubations and storing parts for molecular work. In addition to patch material, plant parenchyma of uncolonized internodes (*n* = 6), typically serving as initial substrate for patches, was sampled for incubations as well. The head space in the glass vials was exchanged by first inducing vacuum using a syringe and then injecting 2 mL of the artificial atmosphere. ^15^N_2_ gas (98 at%) used in incubations was purchased from CAMPRO Scientific (Berlin, Germany, lot # MBBB0968V) and Cambridge Isotope Laboratories (Tewksbury, MA, USA, lot # I-21117A) and tested negative for ^15^N-labelled ammonia gas contaminations using the hypobromite oxidation method [67, 68]. After incubation for 72 h, the vials were opened and patches were dried at 60 °C. The ^15^N/^14^N isotopic ratios were determined using an elemental analyzer (EA 110; CE Instruments, Milan, Italy) coupled to an isotope ratio mass spectrometer (DELTA Plus; Finnigan MAT, Bremen, Germany) in Vienna, Austria. Non-incubated parenchyma (*n* = 4), IPs (*n* = 33), and EPs (*n* = 84) served as natural abundance controls. The measured ^15^N enrichment per sample was calculated to atom percent excess $\left(\mathrm{APE}=\left[\mathrm{at}{\%}_{\mathrm{inc}.}-\mathrm{at}{\%}_{\mathrm{nat}.\mathrm{ab}.}\right]\times \left[\frac{100}{\mathrm{at}{\%}_{{}^{15}{\mathrm{N}}_2\mathrm{gas}}-\mathrm{at}{\%}_{\mathrm{nat}.\mathrm{ab}.}}\right]\right)$. The amount of fixed N was calculated for each sample as N per mg patch dry weight [μg N mg^−1^ dw] $\left(\mathrm{N}=\left[\frac{{\mathrm{APE}}_{\mathrm{sample}}}{100}\right]\times \left[\frac{{\left(\mathrm{Amt}\%\mathrm{N}\right)}_{\mathrm{sample}}}{100}\right]\times 1000\right)$.

### Investigating the diazotrophic community composition

In total, the diazotrophic communities of 33 early colony stages (*A. alfari* (19), *A. constructor* (6), *A. xanthochroa* (8)) and 36 established ant colonies (*A. alfari* (12), *A. constructor* (24)) were investigated (detailed overview in Additional file 1: Table S3). Some *Cecropia* plants were colonized by several foundress queens, each in a spatially segregated domatium. From the early ant colony stage, the single, initial patch was sampled. From 15 established ant colonies, patch material was sampled up to three times throughout the nesting space. As new domatia are formed apically during plant growth, recently created patches were from the top, while older patch material was sampled from lower parts of the plants. Collected patch samples were preserved in RNAlater (Thermo Fisher Scientific, Waltham, MA, USA) and stored at − 20 °C in the field and at − 80 °C after returning to Vienna. Details of the molecular analysis to investigate the diazotrophic community composition can be found in the Additional file 3: supplementary methods. In short, nucleic acid extractions of RNAlater-removed samples were performed according to a bead-beating phenol/chloroform-based protocol [69]. To examine the diazotrophic communities, we amplified the dinitrogenase reductase-encoding gene (*nifH*) [42, 43] using Ueda19F as forward and R6 as reverse primer [70, 71], as these showed the least amplification bias regarding *nifH* homologous genes (*bchX*, *chlL*, *bchL*, *parA*) [44]. To uncover the diazotrophic communities transcribing the *nifH* gene and therefore potentially actively fixing N_2_ at the time of sampling, purified RNA was reverse transcribed into cDNA. As extractions of IPs did not yield sufficient nucleic acids for RNA purification, only EPs were used for investigating the transcribing diazotrophic communities. *NifH* genes and transcripts of the template DNA and cDNA were amplified in a two-step barcoding approach for subsequent Illumina MiSeq sequencing according to Angel et al. [44]. Raw MiSeq amplicon reads were deposited in the NCBI Short Read Archive under the BioProject accession number PRJNA777006 [72] and processed using the pipeline NifMAP [44]. The clustered OTUs (3% radius) were taxonomically classified using BLASTP [73] against the RefSeq database [74]. OTUs were assigned to phylogenetic clusters based on Classification And Regression Trees (CART) [75]. Samples resulting in less than 300 *nifH* reads were discarded. The remaining samples harbored on average around 6000 *nifH* reads amplified from DNA and 1900 *nifH* reads amplified from cDNA.

### Quantitative PCR assays

The abundances of potential diazotrophs in patches of established ant colonies were assessed by quantitative PCR assays (qPCR) targeting the *nifH* gene using the same primer pair as for amplicon sequencing. The total microbial abundances were investigated using primers 515F-mod and 806R-mod [76] targeting the 16S rRNA gene. Ten EPs of both *Azteca* species were analyzed. For quantification of both genes, serially diluted standards (ranging between 10^1^ and 10^6^ copies) were added per reaction plate. Details on the used standards and the performed qPCR assay according to Angel et al. [44] are listed in the Additional file 3: supplementary methods. The *nifH* gene copy number did not have to be corrected, as homologous non-*nifH* reads were not detected in amplicon libraries of the same samples.

The abundance of diazotrophs per EP sample was calculated as the proportion of diazotrophic bacteria (*nifH* copy number) to total bacteria (16S rRNA gene copy number). As copy numbers of both genes might be different in the genomes, this abundance is an estimate. While the 16S rRNA gene copy numbers are known to vary in genomes [77, 78], also additional *nifH* copies can exist in the chromosome, e.g., in *Clostridium pasteurianum* [79], or on a symbiotic plasmid, e.g., in *Bradyrhizobium* sp. DOA9 [80]. Furthermore, every known diazotroph which encodes an alternative V or Fe-only nitrogenase also possesses the canonical Mo nitrogenase [81].

### Statistical analyses

All analyses were performed using R (version 3.5.2 [82];) and were plotted using the package ggplot2 (version 3.3.3 [83];). All functions originated from the package vegan (version 2.5-6 [84];), unless otherwise mentioned.

A Wilcoxon rank sum test (function wilcox.test, package stats, version 3.5.2 [82];) was used to test whether APE differs significantly across patch types and uncolonized parenchyma (pairwise, *p*-value corrected), and whether BNF rates correlate with patch types or ant species within EPs. Kruskal-Wallis rank sum test (function kruskal.test of the package stats) was used to test if BNF rates of IPs correlate with the ant species. The Wilcoxon rank sum test was also used to test for correlations between quantitative abundance data (gene copy numbers and their ratio) and ant species.

For all OTU-based distance matrices, Bray-Curtis dissimilarity measures were used. Permutational multivariate ANOVA (PERMANOVA) was used for variance partitioning analyses (adonis function) [85]. For research question-based data subsetting and computing Shannon alpha diversity indices (function estimate_richness), the phyloseq package was used (version 1.26.1 [86];). For testing correlations of alpha diversity differences with categories (patch type and ant species), the Wilcoxon and Kruskal-Wallis rank sum tests were used. The package microbiome was used for core microbiome analyses (core function, version 1.5.28 [87];). Read counts were converted into relative abundance for community composition analyses. For comparisons between communities of IPs and EPs, as well as EP communities between ant species, biological replicates of the same ant colony (Additional file 1: Table S3) were merged by calculating the average community composition. A PERMANOVA was used for testing correlations between categories (patch type, ant species within patch types, assumed patch age and ant colony within multiple-sampled established ant colonies, and tree specimens within multiple queen-colonized *Cecropia* trees) and variation in community composition. Significant factors were displayed as constrained distance-based redundancy analysis (dbRDA) via the function capscale. To evaluate per ant species if alpha diversity measures of the general diazotrophic and *nifH-*transcribing communities in EPs are significantly varying, the Wilcoxon rank sum tests were used. Analysis scripts are available at https://github.com/mnepel/nitrogen_fixation_arboreal_ant_nests [88].

## Supplementary Information

**Additional file 1: Table S1.** Relative read abundances of the most abundant taxonomic orders per patch type. **Table S2.** Calculating BNF rates in fungal gardens of leaf cutter ants. **Table S3.** Overview on the number of sampled *Cecropia* trees, early stage and established ant colonies.

**Additional file 2: Figure S1.** Alpha diversities in samples due to different patch types of individual *Azteca* species. **Figure S2.** Alpha diversities in patch samples of established ant colonies on *nifH* gene and transcript level. **Figure S3.** *NifH* transcribing community on genus level in selected patch samples of established *Azteca alfari* and *A. constructor* colonies with measured APE higher than the overall median. **Figure S4.** Oxygen gradients through patches of established *Azteca* colonies.

**Additional file 3: Supplementary methods.** Detailed information on molecular analysis and performed qPCR assays, calculating BNF rates in fungal gardens of leaf cutter ants, evaluating the potential ecological role of BNF for ant colony growth, and oxygen profiling through established patches.

## Data Availability

The raw sequencing reads were deposited in the NCBI Short Read Archive under the BioProject accession number PRJNA777006 [72].

## Abbreviations

APE: Atom percent excess
BNF: Biological N fixation
C: Carbon
dw: Dry weight
EP: Established patch
IP: Initial patch
N: Nitrogen
OTU: Operational taxonomic unit
Pa: Parenchyma
qPCR: Quantitative PCR
