# Supplementary material for: Nitrogen fixation by diverse diazotrophic communities can support population growth of arboreal ants
Source: BMC Biol. 2022 Jun 9;20:135. doi: 10.1186/s12915-022-01289-0 (PMC9185989; doi:10.1186/s12915-022-01289-0)
Supplement: Supplementary file 1 — Additional file 1: Table S1. Relative read abundances of the most abundant taxonomic orders per patch type. Table S2. Calculating BNF rates in fungal gardens of leaf cutter ants. Table S3. Overview on the number of sampled Cecropia trees, early stage and established ant colonies. [file 12915_2022_1289_MOESM1_ESM.pdf]

Additional file 1

Accompanying Nepel, M., Pfeifer, J., Oberhauser, F.O., Richter, A., Woebken, D., Mayer, V.E., Nitrogen fixation by diverse diazotrophic communities can support population growth of arboreal ants. BMC Biol 2022.

This PDF file contains

- Tables S1-S3
    - Table S1 – Relative read abundances of the most abundant taxonomic orders per patch type.
    - Table S2 – Calculating BNF rates in fungal gardens of leaf cutter ants.
    - Table S3 – Overview on the number of sampled *Cecropia* trees, early stage and established ant colonies.

Table S1: Relative read abundances of the most abundant taxonomic orders per patch type. For each order the average relative read abundance per sample and the range across samples are listed per patch type.

	Initial patches		Established patches	
	average [%]	range [%]	average [%]	range [%]
Bacteroidia				
Bacteroidales	0.01	[0-0.3]	3.1	[0-54.6]
Cyanobacteria				
Nostocales	10.6	[0-89.6]	1.7	[0.1-26.9]
Firmicutes				
Clostridiales	2.9	[0-31.5]	7.9	[0.1-55.5]
Alphaproteobacteria				
Rhizobiales	37.1	[0.3-97.8]	25.0	[2.4-75.5]
Rhodospirillales	1.5	[0-11.5]	12.3	[0.4-61.7]
Betaproteobacteria				
Burkholderiales	4.0	[0-91.5]	8.5	[0.3-88.7]
Rhodocyclales	17.1	[0-89.4]	12.4	[0.3-85.0]
Gammaproteobacteria				
Enterobacterales	24.3	[0.4-99.3]	11.9	[0.4-63.9]
Opitutae				
Opitutales	0.5	[0-19.1]	4.7	[0-74.9]

Table S2: Calculating BNF rates in fungal gardens of leaf cutter ants, via at% and APE. Delta ^{15}N ($\delta\,^{15}N$) measurements of incubated samples and natural abundance controls were published in Pinto-Tomas *et al.* [48].

	$\delta\,^{15}N$ nat.ab. (‰)	$\delta\,^{15}N$ inc. (‰)	at%^{15}N nat.ab. (%)	at%^{15}N inc. (%)	APE (%)	fixed N per sample dry weight for the incubation period [µg N µg^{-1}]	fixed N per sample dry weight per day [µg N g^{-1} d^{-1}]
fungal.garden.week1	2.28	3.19	0.367305	0.367637	0.000332	1.16E-07	1.66E-02
fungal.garden.week2	1.82	3.32	0.367137	0.367684	0.000548	1.92E-07	1.37E-02

Table S3: Overview on the number of sampled *Cecropia* trees, early stage and established ant colonies, used for investigation of diazotrophic community composition. As mentioned in the main manuscript, for certain research questions and analyses only subsets were used, or biological replicates (like repeatedly sampled established ant colonies) were merged. IP refers to "initial patch", EP to „established patch".

	**No. of *Cecropia* trees**	***Azteca alfari* colonies**	***Azteca constructor* colonies**	***Azteca xanthochroa* colonies**	**Total no. of patches**	**Comment**
**Early ant colony stage**						
one IP sampled per *Cecropia* tree	9	7	1	1	9	only one internode per tree was colonized by a foundress queen
> 1 IPs sampled per *Cecropia* tree	9	13	4	7	24	multiple internodes per tree were colonized by one foundress queen each
Sum		20	5	8	33	
**Established ant colonies**						
one EP sampled per *Cecropia* tree	21	7	14	-	21	patch material was only sampled once per established ant colony
> 1 EP sampled per *Cecropia* tree	15	4 col. (10 EPs)	11 col. (25 EPs)	-	15 col. (35 EPs)	patch material was sampled up to three times throughout the nesting space
Sum		11	25	-	36 col. (56 EPs)	
