# Supplementary material for: Nitrogen fixation by diverse diazotrophic communities can support population growth of arboreal ants
Source: BMC Biol. 2022 Jun 9;20:135. doi: 10.1186/s12915-022-01289-0 (PMC9185989; doi:10.1186/s12915-022-01289-0)
Supplement: Supplementary file 2 — Additional file 2: Figure S1. Alpha diversities in samples due to different patch types of individual Azteca species. Figure S2. Alpha diversities in patch samples of established ant colonies on nifH gene and transcript level. Figure S3. NifH transcribing community on genus level in selected patch samples of established Azteca alfari and A. constructor colonies with measured APE higher than the overall median. Figure S4. Oxygen gradients through patches of established Azteca colonies. [file 12915_2022_1289_MOESM2_ESM.pdf]

Additional file 2

Accompanying Nepel, M., Pfeifer, J., Oberhauser, F.O., Richter, A., Woebken, D., Mayer, V.E., Nitrogen fixation by diverse diazotrophic communities can support population growth of arboreal ants. BMC Biol 2022.

This PDF file contains

- Figures S1 to S4
    - Fig. S1 – Alpha diversities in samples due to different patch types of individual Azteca species.
    - Fig. S2 – Alpha diversities in patch samples of established ant colonies on *nifH* gene and transcript level.
    - Fig. S3 – *NifH* transcribing community on genus level in selected patch samples of established *Azteca alfari* and *A. constructor* colonies with measured APE higher than the overall median.
    - Fig. S4 – Oxygen gradients through patches of established *Azteca* colonies.

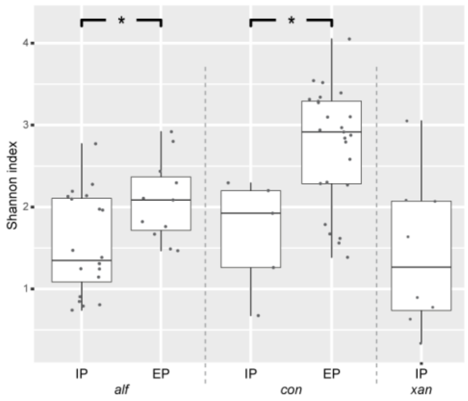

Fig. S1: Significant variations of alpha diversities (Shannon index) due to different patch types of individual ant species. On the x-axis samples are grouped per patch types (initial patch, IP; established patch, EP) and ant species (*alf*: *Azteca alfari*; *con*: *A. constructor*; *xan*: *A. xanthochroa*). Dots represent diversity values of single samples (significance levels: P=0.021; P=0.012, respectively).

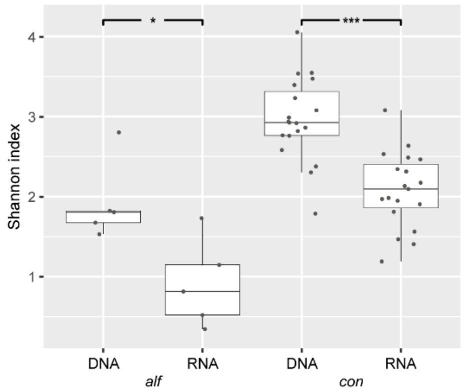

Fig. S2: Significant decrease of diazotrophic alpha diversities in patch samples of established ant colonies from general diazotrophic to *nifH* transcribing communities. On the x-axis samples are grouped by nucleic acid type (DNA, RNA) and ant species (*alf*: *Azteca alfari*; *con*: *A. constructor*). The y-axis shows the Shannon index. Dots represent diversity values of single samples. (significance level: * P=0.036; *** P<0.001)

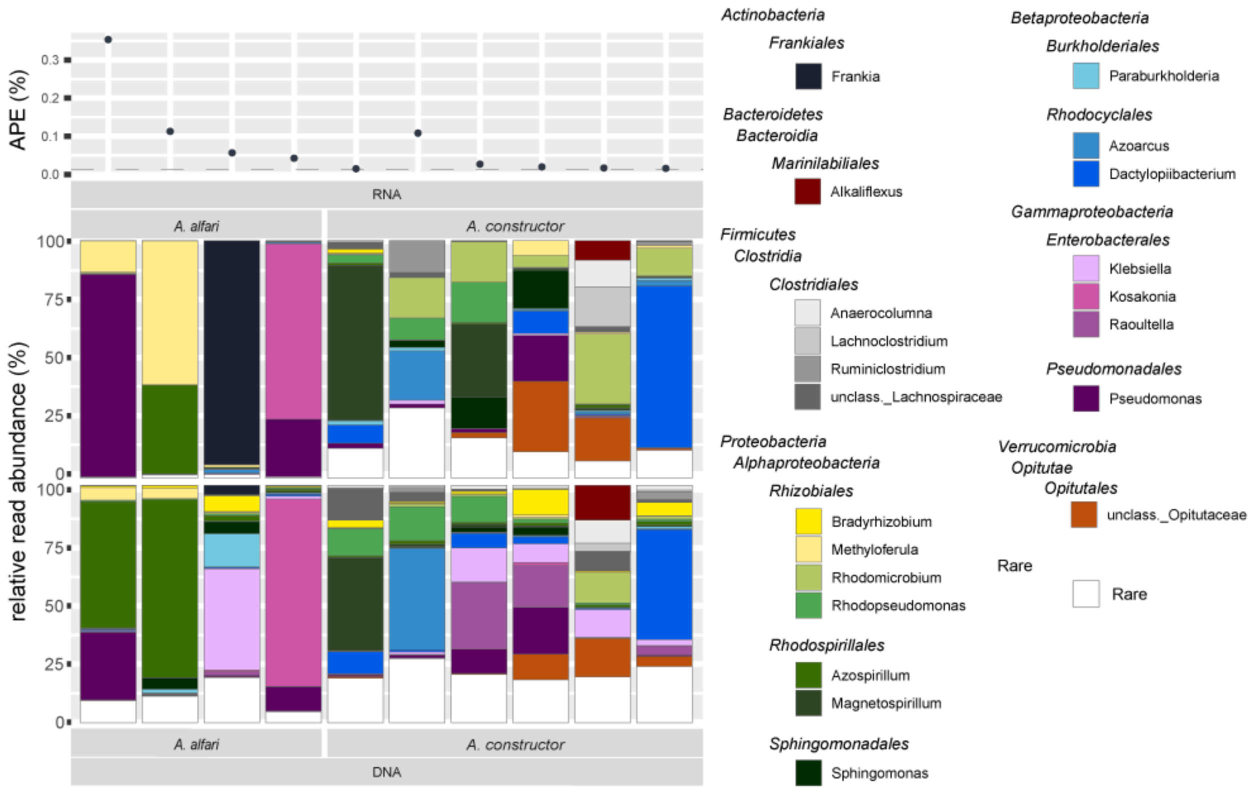

Fig. S3: High heterogeneity of the *nifH* transcribing community in selected EP samples of *Azteca alfari* and *A. constructor* at the time of sampling with measured APE higher than the overall median. Taxonomic assignments of OTUs are summarized on the genus level based on BLASTP analysis. On the x-axis every bar represents the diazotrophic community (bottom), the actively transcribing community (middle) of one EP sample, grouped by ant species. The y-axis shows the relative read abundance of taxonomic orders. The top graph shows the corresponding APE after sample incubations. The dashed light grey line denotes the overall median APE of all measured EP samples.

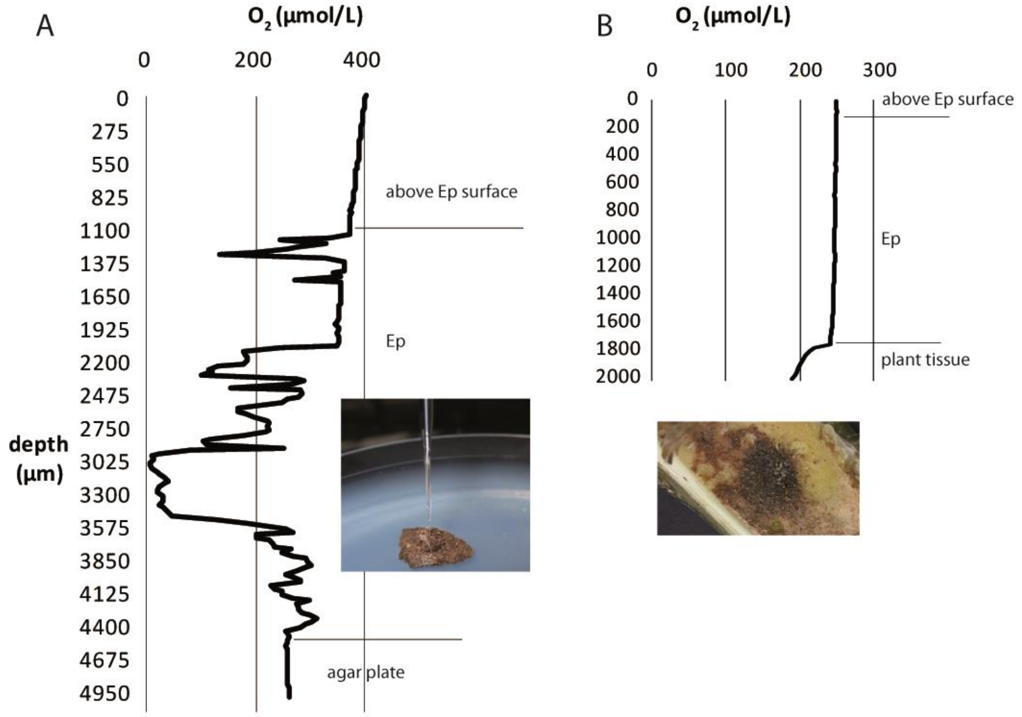

Fig. S4: Oxygen gradients through patches of established *Azteca* colonies. (a) The patch of *A. constructor* was placed on an agar plate and pierced through while oxygen profiling. (b) The lawn-like patch of *A. alfari* was kept in the plant internode to retain the natural patch texture. The x-axis depicts the oxygen concentration and the y-axis denotes the depth profiling through the patch samples.
