# Supplementary material for: Nitrogen fixation by diverse diazotrophic communities can support population growth of arboreal ants
Source: BMC Biol. 2022 Jun 9;20:135. doi: 10.1186/s12915-022-01289-0 (PMC9185989; doi:10.1186/s12915-022-01289-0)
Supplement: Supplementary file 3 — Additional file 3: Supplementary methods. Detailed information on molecular analysis and performed qPCR assays, calculating BNF rates in fungal gardens of leaf cutter ants, evaluating the potential ecological role of BNF for ant colony growth, and oxygen profiling through established patches. [file 12915_2022_1289_MOESM3_ESM.pdf]

Additional file 3

Accompanying Nepel, M., Pfeifer, J., Oberhauser, F.O., Richter, A., Woebken, D., Mayer, V.E., Nitrogen fixation by diverse diazotrophic communities can support population growth of arboreal ants. BMC Biol 2022.

This PDF file contains

- Supplementary methods
  - Detailed information on molecular analysis to study the diazotrophic community composition
  - Details on the performed qPCR assay
  - Calculating BNF rates in fungal gardens of leaf cutter ants
  - Evaluating the potential ecological role of BNF for ant colony growth
  - Oxygen profiling through established patches

Supplementary methods

Detailed molecular analysis to study the diazotrophic community composition

Prior to nucleic acid extractions, samples were transferred into lysing matrix E (Thermo Fischer Scientific) and RNAlater was removed by two rounds of washing using centrifugation at 4 °C and 14,000 g for 1 min, removing supernatant and adding phosphate buffer (pH 8.0). The DNA concentrations of extracted nucleic acids were measured using Quant-iT PicoGreen dsDNA Assay (Thermo Fischer Scientific). To investigating the *nifH* gene transcripts, 1 µg DNA of EP nucleic acid extracts was digested by incubating it two times for 30 min at 37 °C, each time in the presence of 1 µL RNaseOUT and 2 µL of Turbo DNase (both from Thermo Fischer Scientific). The RNA was subsequently purified using GeneJET RNA Cleanup and Concentration Micro Kit (Thermo Fischer Scientific) and eluted in nuclease free water. The RNA concentration was measured using the Quant-iT RiboGreen assay (Thermo Fisher Scientific). To ensure an effective DNA digestion, the same two step amplification protocol used for DNA samples was also performed with purified RNA. Only if *nifH* gene amplification failed in RNA, the DNA digestion was sufficient. Purified RNA (50 ng) was reverse transcribed into cDNA by using Superscript IV and Random hexamer primers (both from Thermo Fisher Scientific). *NifH* genes and transcripts were amplified in standardized 10 ng template DNA and 1 µL of template cDNA. At least one negative control sample using water instead of template DNA/cDNA was included in every performed PCR run to ensure no contamination due to consumables. Illumina Truseq library preparation and MiSeq sequencing of *nifH* DNA and cDNA was performed by Microsynth (Balgach, Switzerland) in the 2 × 300 cycle configuration using the MiSeq Reagent kit V3 (Illumina, San Diego, CA, United States).

Details on the performed qPCR assay

Cloned target gene fragments of *E. coli* for the 16S rRNA gene and of *Didymococcus colitermitum* TAV2 for the *nifH* gene were used as standards. Each qPCR reaction was 20 µl in volume and contained 10 µL of iQ SYBR Green Supermix already including 3 mM $MgCl_2$ (Bio-Rad, Hercules, CA, USA), BSA (Thermo Fisher Scientific) in a final concentration of 0.8 µg/µL and 1.4 µM per *nifH* or 0.5 µM per 16S rRNA primer and 0.8 ng DNA template. Every performed qPCR run included three negative controls using water instead of template DNA to ensure no contamination due to consumables. Quantitative PCR was performed on a C1000 Touch thermocycler equipped with a CFX96 Real Time System (Bio-Rad).

Calculating BNF rates in fungal gardens of leaf cutter ants, via at% and APE

To allow us the direct comparison between BNF activity in leaf cutter ants' fungal gardens and *Azteca* ant-built patches, δ ^{15}N measurements from Pinto-Tomás and colleagues [48] were transformed to BNF rates to account for differences in N content and incubation period. Based on the published δ ^{15}N measurements and N content of fungal gardens (3.5%) [48], we calculated at% ^{15}N (of incubated samples and natural abundance controls) including the natural abundance of ^{15}N in the atmosphere ($^{15}N/^{14}N$=0.0036782) (Additional file 1: Table S2).

$$\left(at\%\ ^{15}N = \left[\frac{(^{15}\mathrm{N}/^{14}\mathrm{N})_{\mathrm{atmosphere}} \times \left(\frac{\delta^{15}N}{1000} + 1 \right)}{1 + (^{15}\mathrm{N}/^{14}\mathrm{N})_{\mathrm{atmosphere}} \times \left(\frac{\delta^{15}N}{1000} + 1 \right)} \right] \times 100 \right)$$

We further assessed APE and calculated BNF rates in µg N g^{-1} (dw) d^{-1} for fungal gardens. The estimated average daily BNF rate was 0.015 µg N g^{-1} (dw) d^{-1}.

Evaluating the potential ecological role of BNF for ant colony growth

To evaluate how much N can be fixed in ant colonies and if these amounts could be beneficial for ants, we focused on the maximum fixation rates. These rates represent the assessed BNF capacities in our experiments. The average dry weight (DW) of IPs in single, queen-colonized plant internodes is 5.5 mg (n=40), whereas the total DW of EPs collected from 10-15 m high *Cecropia* trees amounts to 285 mg in *A. alfari* colonies (n=2) and 2,200 mg in *A. constructor* colonies (n=3). This results in a maximum of 0.12 µg daily fixed N in IPs, and up to 9.32 µg N d^{-1} in EPs of *A. alfari* and up to 139.59 µg N d^{-1} in EPs of *A. constructor*. During the ant's development, at least 14 µg N per larvae is needed - which resembles the average amount of total N in *Azteca* worker ants (n=8; measured by same mass spectrometry as patch samples). The daily fixed N corresponds to a worker ant's N content of approximately 0.8% (early founding stage), 66% (*A. alfari* colony) and 900% (*A. constructor* colony). The distinct difference between both ant species is solely based on the different amount of patch biomass in established ant colonies.

Oxygen profiling through established patches

Intact patches of established *A. constructor* colonies were collected in the field, put into a small Petri dish with a moist filter paper to keep the humidity and subsequently transported to Vienna. As patches of established *A. alfari* colonies are lawn-like and therefore hardly detachable in an intact form, small cut *Cecropia* stems were transported to Vienna. For oxygen profiling through patches the microsensor (OX-25) of Unisense (Aarhus, Denmark) was used. EPs of *A. constructor* were placed on an agar plate to facilitate piercing through the whole Ep, whereas EPs of *A. alfari* were kept in the cut-open stem piece and pierced through carefully until reaching the plant tissue (Additional file 2: Fig. S4).
